# Transient locking of the hook procures enhanced motility to flagellated bacteria

**DOI:** 10.1038/s41598-017-16562-4

**Published:** 2017-11-27

**Authors:** Ismaël Duchesne, Tigran Galstian, Simon Rainville

**Affiliations:** Department of Physics, Engineering Physics and Optics, Center for Optics, Photonics and Lasers, Laval University, Quebec city, G1V 0A6 Canada

## Abstract

Flagellated bacteria often proliferate in inhomogeneous environments, such as biofilms, swarms and soil. In such media, bacteria are observed to move efficiently only if they can get out of “dead ends” by changing drastically their swimming direction, and even to completely reverse it. Even though these reorientations are ubiquitous, we have only recently begun to describe and understand how they happen. In the present work, we visualized the flagella of bacteria swimming in a soft agar solution. The surprising observation that the filaments do not rotate while being flipped from one side of the cell to the other suggests that reversals are driven directly by the motor rather than by the thrust created by the rotating filament. This was confirmed by observing bacteria in a liquid crystal, where the linear movement of bacteria greatly simplifies the analysis. These observations suggest that the reversal and reorientation processes involve a temporary locking of the flagellum’s hook, which is the normally flexible joint between the rotary motor and the long helical filament that propels the cell. This newly described locked-hook mode occurs only when the motor switches to a clockwise rotation. That correlates with other phenomena that are triggered by a switch in one direction and not the other.

## Introduction

**Figure 1:**
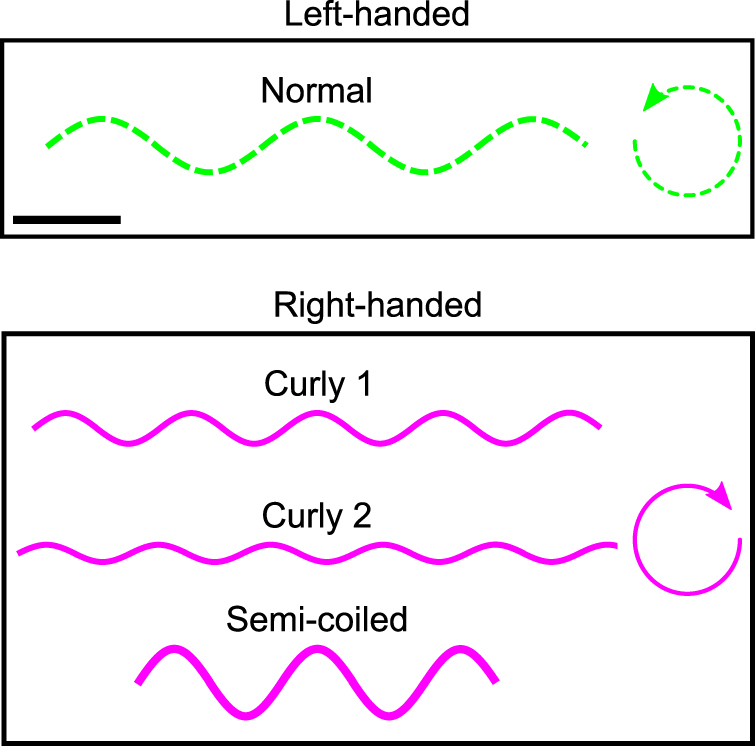
Schematic representation of four typical filament conformations observed in our experiments. The normal filament is left-handed and rotates CCW. Curly and semi-coiled filaments are right-handed and rotate CW. The black scale bar in the upper box measures 1 *μ*m.

Studying bacterial motility is crucial to understand how bacteria can colonize biological tissues. Bacteria can move using a number of different strategies. A very common one is the flagellar motor that allows bacteria to swim in liquid environments by rotating a long extracellular filament^1^. *Salmonella* and *E. coli* strain are two well-known gram-negative flagellated bacteria. These bacteria have one or few flagella composed at their base of a rotary motor anchored in the membrane. A flexible universal joint (called the hook) allows the transmission of the rotation from the motor to a semirigid helical filament that propels the bacteria^1,2^. The hook is a complex assembly (55 nm long) of identical proteins (FlgE) that must change their relative positions during one rotation to allow the axes of rotation of its two ends to differ^3,4^. Finally, the flagellar filament has a diameter of around 20 nm and a length of several micrometers. The motor can rotate either in the clockwise (CW) or counterclockwise (CCW) direction (as seen by an observer looking towards the cell body). In isotropic liquids, the CCW rotation of the motors is associated with bacteria swimming in a straight line (runs) while switching to CW rotation generally induces changes in direction (tumbles). Depending on the direction of rotation and several other parameters, the filament can take one of 11 polymorphic conformations with a specific helicity (handedness), pitch and radius^5,6^. In this work, we observed four main conformations, one left-handed and three right-handed (Fig. 1). When the motor rotates CCW, the filament has the normal conformation and when it rotates CW, the conformation is semi-coiled or curly (either curly 1 or 2, which differ by their amplitude and spatial pitch). Throughout this work, it is the identification of theses conformations that enables us to infer the direction of rotation of the filament in each image of our movies.

Bacteria often swim in inhomogeneous restrictive environments, such as biofilms, tissues or soil, where they encounter many obstacles and dead ends^7,8^. To navigate efficiently in these media, flagellated bacteria have to be able to drastically change their swimming direction and even reverse their movement^9–13^. It is also well established that bacteria have to be able to switch the direction of rotation of their motors to move efficiently in soft agar, a fluid-filled porous medium common in microbiology^14–16^. However, the detailed mechanism behind motility in such restrictive environments is unknown. The goal of this work is to investigate this crucial aspect of bacterial motility at the level of the filament.

By observing fluorescently labeled filaments of bacteria swimming in agar, we noted that a reversal or drastic reorientation of bacteria occurs only when one filament is driven by the motor to a new position and orientation. This transition only happens when the motor switches from a CCW to a CW rotation (denoted a CCW–CW switch). To isolate the mechanical process behind these events, we used a liquid crystal (LC) that can linearize the movement of the filament and the bacterium^12,17–19^. From our observations with this elegant tool, we hypothesize that reversal and reorientation events are caused by a momentary locking of the hook.

## Results

### Escapes of single flagellated bacteria in agar solution

To simplify the observations and analysis, we conducted these experiments with a strain of *Salmonella* bacteria that have a single flagellum. The strain EM800 has exactly the same flagellar motor as the wild type, but instead of having multiple flagella randomly placed on its body, it forms only one flagellum that can be fluorescently labeled^20^. We incorporated a warm solution (about 45 °C) of 0.3% agar containing this strain of bacteria in a custom chamber. Before each experiment, the chamber was cooled down (to room temperature) for 5 minutes, so that the agar matrix can form. We recorded around 70 videos (each 10 seconds at 193 fps) of bacteria swimming in the maze formed by the agar. As previously observed for multi-flagellated bacteria^14^, the swimming tracks are composed of relatively straight runs until bacteria get stuck in a dead end formed by the agar. After several seconds, they escape by drastically changing the orientation of their body. In some cases, the body maintains the same orientation, but the direction of motion is reversed.

**Figure 2:**
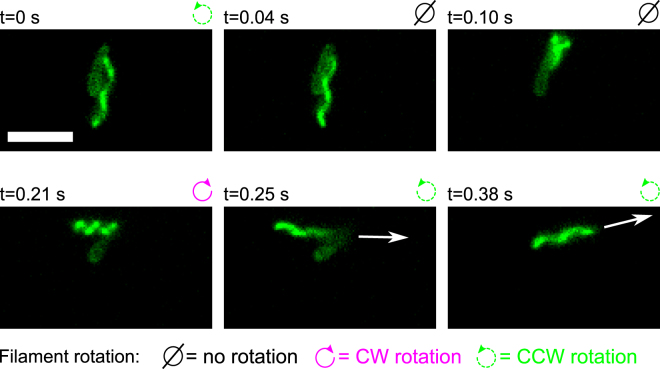
Sequence of images taken from movie S1A (see also movies S1*B*–*E*) that show a bacterium escaping from an obstacle (not visible in images) in soft agar. The time stamp of the frame is shown above each image. The cell is at rest in every image except in the last two where the white arrows indicate the direction of the displacement of the bacterium’s body. The mean speed of the bacterium during straight run was of 13±2 *μ*m/s. The white scale bar at the bottom left of the first image measures 5 *μ*m. See text for more details.

To understand how bacteria can escape from dead ends, we observed the filaments of bacteria stuck in the agar matrix. We paid particular attention to the conformation of the filament to identify the direction of rotation of each filament (see the subsection Acquisition of Methods below). We noticed that escapes arise mainly (84 occurrences out of 86) when the flagellar motor switches its direction of rotation from CCW to CW. These switches triggered a reorientation of the filament and body of the bacterium. Indeed, the filament conformation before its reorientation was always normal (CCW rotation) and immediately after the event, it was curly (1 or 2) or semi-coiled (CW rotation). We can see a typical example of these reorientations in Fig. 2 and movie S1*A* (see also movies S1 *B*–S1*E*). At *t* = 0 s the bacterium is stuck in an agar cluster while its filament rotates CCW and has a normal conformation. At *t* = 0.04 s, the rotation of the filament stops (presumably after a CCW–CW switch followed by the unwinding of the hook^6^). At *t* = 0.10 s, the orientation of the filament changes while its rotation (about its axis) is still stopped. This reorientation starts from the proximal end of the filament and appears to be the result of a rotation of the filament around the axis of the motor. Indeed, when the body of the bacterium is free to rotate, this motion induces a rotation of the body in the opposite direction (as can be seen in frames 125–150 of movie S1*B*). After the reorientation of the filament, at *t* = 0.21 s, its conformation becomes semi-coiled and the CW rotation starts to be visible while the body continues to reorient. After the motor switched back to a CCW rotation, at *t* = 0.26 s, the filament returns to a normal conformation while the new spacial orientation of the cell body is conserved. We note that the reorientation of the swimming direction is first driven by the drastic reorientation of the filament (from *t* = 0.04 s to *t* = 0.21 s) and then, by the thrust created by the rotating filament (from *t* = 0.21 s to *t* = 0.38 s) since the filament and the bacterium are not aligned. We observed 2 escapes (out of 86) where a conformation change was not perceptible after the reorientation of the filament (corresponding to the step shown in the bottom left panel of Fig. 2). Since all the other steps were identical, we think that these escapes were also triggered by CCW–CW switches, except that the motor switched back to a CCW rotation too quickly to allow the conformation change.

**Figure 3:**
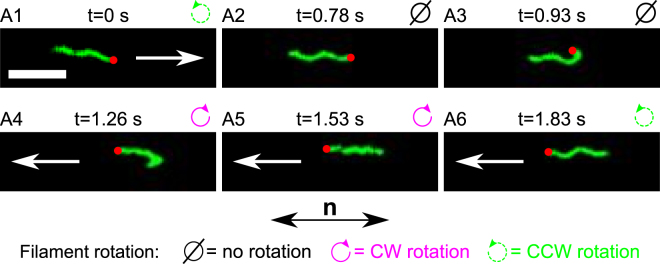
Sequence of images taken from movie S2*A* (see also movies S2 *B* and S2*C*) showing an example of the locked hook during a reversal of the filament (reorientation of 180°). Above each image, we show the step label corresponding to the schematic drawing in Fig. 5, the time stamp of each movie frame, and the direction of rotation of the filament. The white arrows indicate the direction of the displacement of the bacterium (the mean speed of single flagellated bacteria was 1.7 ± 0.9 *μ*m/s). There is no arrow in A2 and A3 because the cell body is at rest. The red dot identifies the base of the filament. The white scale bar at the bottom left of the first image measures 5 *μ*m. The bottom arrow labeled **n** indicates the orientation of the anisotropy axis of the LC (identical in all frames).

Interestingly, CW–CCW switches seem unable to trigger the drastic reorientation of the filament needed to escape from obstacles. Indeed when a CW–CCW switch occurs, the filament’s orientation does not change much, as can be seen in the last two frames (A5 and A6) of Fig. 3. In addition, the reorientation triggered by CCW–CW switches cannot come from the thrust created by the filament since it does not rotate during the reorientation. Hence, this event must arise from a mechanical process induced directly by the motor. However, two experimental challenges limit our ability to observe precisely this process in agar: the filament rarely remains in the focal plane during a reorientation, and it can interact with inhomogeneities in the agar matrix. As a tool to bypass these limitations and obtain better observations of filaments’ reorientations, we used a medium that can linearize the movement of the bacterium: a lyotropic liquid crystal (LC)^12,17–19^.

### Reversals of bacteria swimming in a liquid crystal

Lyotropic LCs reproduce the anisotropic elastic properties of many biological environments such as mucus, synovial fluid and biofilms^21–24^. Recent studies have revealed that bacteria swim along linear trajectories in such media^12,17–19^. These one-dimensional movements include sudden reversals in the swimming direction (without reorientation of the cell body). The same phenomenon was previously observed for bacteria swimming in restrictive (confined) environments^9–11^, for swarming bacteria^25^ and, as described above, in soft agar. By using the LC host, we are hoping to describe the mechanical process that produces the remarkable reorientations of bacteria in all these cases.

We recorded around 100 videos (on average 10 seconds each at 30 fps) of bacteria swimming in a solution of 12 wt% disodium cromoglycate (DSCG) in deionized (DI) water. At that concentration, the molecular aggregates of DSCG form an anisotropic elastic liquid by naturally aligning^26^. As previously observed for strains of bacteria with multiple filaments^12,17–19^, the trajectories of EM800 bacteria were confined to a straight line (along the direction of the anisotropy axis of the LC), and they occasionally stopped and then reversed their swimming direction. To verify if the LC also constrained the dynamics of the filaments, we first focused our attention on the filaments’ orientation. As expected, both the filaments and the cell bodies were always aligned with the director **n** (the local macroscopic anisotropy axis) of the LC (see Fig. 3 and movies S2*A*–*C*, S3*A*–*C* and S4*A*–*C*). This is mainly due to the elastic forces created by the LC, but also to the reduced viscosity in the direction of **n** ^17,19^. Together, these two effects imply that the propulsive force is exerted exclusively in the direction of **n**, and this explains why bacteria move only in one dimension (rather than simply having one preferred direction like non-motile diffusing objects)^19,27^.

**Figure 4:**
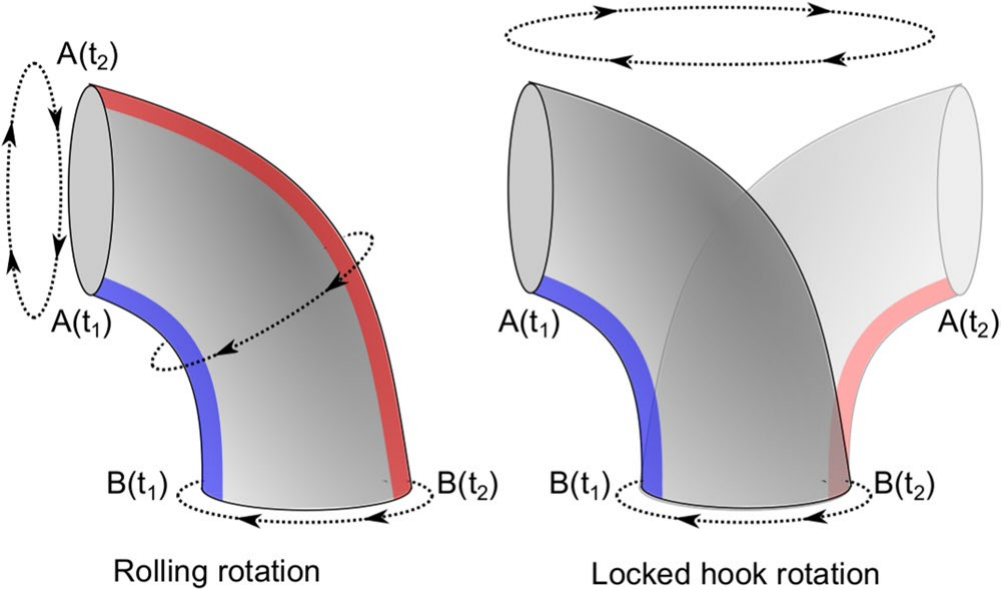
Schematic representation of two possible rotation modes of the hook. The left figure shows the traditional rolling rotation, as a universal joint to transfer the rotation of the motor (which would be at the bottom on the schematic) to the filament (on the left). In this mode, the distance between each protein that composes the hook is modulated periodically to enable a rotation around a stable structure (here a bend to the left)^3,4^. The right figure shows the “locked-hook” rotation, where the entire hook rotates around the axis of the driving motor as a rigid structure. In both figures, the proteins along the blue strip must distribute along the red strip after half a turn.

**Figure 5:**
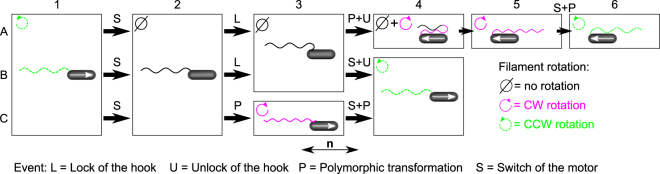
Description of the three typical scenarios that can happen after a CCW–CW switch. Case *A* represents a complete reversal transition of the filament, case *B* corresponds to an incomplete reversal and case *C* shows a continuous run. The black arrows (pointing right) represent short events (below our temporal resolution of 33 ms), while the boxes depict longer steps. The white arrows inside the cell bodies show their displacement direction. When there is no arrow, the bacterium is at rest. The bottom arrow labeled **n** indicates the orientation of the anisotropy axis of the LC. See text for detailed explanations.

Our videos also show the conformation of the fluorescent filaments when their motor switches its direction of rotation. After a CW–CCW switch, the filament keeps its spatial orientation (“behind” the bacterium) while its conformation changes from curly (1 or 2) to normal (Fig. 1). Since these two conformations have opposite helicity, the filament then continues to push the cell in the same direction. This same behavior was observed in agar solutions. In contrast, when a CCW–CW switch occurs (let us call this “case *A*”), we discovered that the hook becomes rigid, it locks its rotation such that it no longer rotates around itself, but around the axis of rotation of the motor, as illustrated in Fig. 4. This changes the orientation of the base of the filament as shown on the panel A3 of Fig. 3 and Fig. 5. This corresponds to the mechanical process that we were looking for in agar solutions. The full sequence of observed events is as follows: initially the bacterium swims (from left to right) with its filament rotating CCW (step A1). At t = 0.78 sec, the filament stops rotating and the bacterium stops moving (step A2). Approximately 0.15 sec later (at t = 0.93 sec), we see that the filament is dragged from the “back” of the bacterium to its “front” (step A3). While the hook reorients the base of the filament, the latter is dramatically bent on itself (with an average radius of curvature of 0.4 ± 0.1 *μ*m). Between the steps A3 and A4, the base of the filament undergoes a polymorphic transformation from normal to curly, and the proximal end of the filament starts to rotate CW while its distal end winds up behind the bend (without rotating) (step A4). At that point, the movement of the cell body restarts in the opposite direction (from right to left). When the entire filament has wound up “behind” the cell, the bacterium runs with its filament in a curly conformation for a short period of time (step A5). Finally, after a CW–CCW switch and a polymorphic transformation (from curly to normal), the bacterium is back to its “initial state” except that it swims in the opposite direction (step A6).

Given that both the filament and the cell body are stopped while the filament moves from one side to the other (in steps A2–A3), we conclude that the flagellar motor slowly rotates a rigid hook (during approximately 0.07 sec.) to drag the filament from one side of the cell to the other. To the best of our knowledge, this is the first report of a hook-locking process. We confirmed that this process is also responsible for the reorientation and reversal events in agar solution since same steps are observed in both media. In addition, the rigid rotation of the filament axis around the motor explains why the body rotates in the opposite direction during reorientation events. As mentioned before, soft agar solutions are an example of confined environments since they are composed of a maze of agar filled with a liquid. Thus, our observations could presumably apply to any restrictive environment. Furthermore, we think that the locked hook must play a critical role in the standard tumble events that are central to purposeful bacterial motility (see Discussion below).

**Figure 6:**
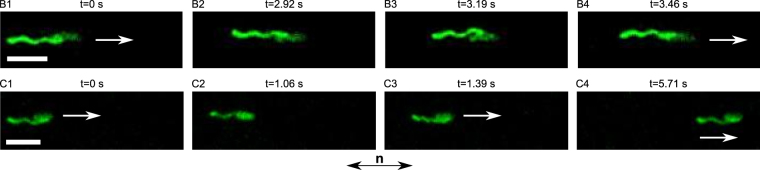
Sequences of images taken from movies S3 *A* and S4*A* (see also movies S3 *B* and S3*C* and S4*B* and S4*C*) showing the cases *B* and *C* described in Fig. 5. Above each image are shown the step number corresponding to the schematic drawing in Fig. 5 and the time stamp of each movie frame. The white arrows indicate the direction of the displacement of the bacterium (the mean speed of single flagellated bacteria was 1.7 ± 0.9 *μ*m/s). When there is no arrow, the bacterium is at rest. The white scale bars measure 5 *μ*m. The bottom arrow labeled **n** describes the orientation of the anisotropy axis of the LC.

Our observations also show that not every CCW–CW switch of the motor triggers a reversal of the filament (67 reversals out of 146 switches: 46%), so it seems that the CCW–CW switches are required but not sufficient to create the hook-locking event. Indeed, two additional scenarios (denoted *B* and *C* below) were observed in our videos following a CCW–CW switch. All three cases are schematically represented in Fig. 5 and movie frames for cases *B* and *C* are shown in Fig. 6. The first steps of the three cases are the same. In step 1, the bacterium swims with a normal filament (CCW rotation). In step 2, after a CCW–CW switch, the filament stops for a short period of time (around 30 ms). In the agar case, this downtime is probably due to the unwinding of the hook^28^. The case *B* corresponds to an incomplete reversal, and was observed 14 times out of 146 switches. Like in case *A*, the hook locks in step B3 and the motor’s rotation starts to reorient the proximal end of the filament. However, the motor switches back to CCW before the base of the filament has rotated by 90°, so that the hook unlocks and the normal filament comes back to its initial side (step B4), rotating CCW just like it was doing in step B1. Finally, the case *C* could be called a “continuous run” (65 occurrences on 146 switches). When the hook has finished unwinding (and before it has the chance to lock), the filament undergoes a polymorphic transformation from normal to curly, starting from its proximal end (step C3). The filament’s rotation then restarts in CW direction so that the cell moves to the right, as it was before. Later on, the motor switches back to CCW and, nearly simultaneously, the filament undergoes a polymorphic transformation back into the normal conformation. The system is then back into its initial state (step C4 is the same as C1).

### Other observations

To test whether the locked hook was specific to the *Salmonella* strain, we also studied the filaments behavior of *E. coli* strain HCB1737^29^. These bacteria have multiple filaments (typically 4–6) that can also be labeled with fluorophores. In this strain also, the hook often locked when a CCW–CW switch occurred, causing the filament to flip to the opposite side of the cell body (see section S1, Fig. S1 and movies S5A and S5B for more details).

As we have seen above, the probability that the hook locks after a CCW–CW switch in single flagellated bacteria, is around 56% (cases A and B together). With the current dataset, we cannot distinguish whether that locking probability reflects slight differences in the structure of individual hooks, or it depends on variations in external conditions. However, we did observe multiple switches in the same cell, and there was no large systematic difference between the locking probability of one cell vs another. In other words, an individual cell exhibits different behaviors (locking or not) on successive switches. You can see behaviors corresponding to cases *A* and *C* in movies S2*B* and S4*C*, to cases *A* and *B* in movies S2*C* and S3*C* and behaviors corresponding to case *A* and a 360° reorientation in movie S7*A*. This makes the possibility of a structural difference (e.g. a mutation in the hook protein FlgE) less likely. We also looked for a relation between hook-locking events and rotation speed or filament length, and we concluded that, unlike other flagellar mechanisms that have been shown to depend on the torque generated by the motor^30–32^, the locking probability of the hook appears to be torque independent (see section S2).

## Discussion

Looking at the shape and dynamics of fluorescently labeled filaments of bacteria swimming in restrictive environments, we have been led to propose that the hook can momentarily lock to impose a change in the orientation of a filament. At first glance, the observations reported here seem to be explained by the polymorphic transformations of the filament triggered by a switch in the motor rotation as described by Kuhn *et al*.^13^ for a different strain. Indeed, these transformations start from the base of the filament and propagate along the entire filament until it has completely changed its shape. At the junction between the two polymorphic forms, there is a bend of the filament of around 90°, but only when the motor switches to a CW rotation^6^. The similarities between this phenomenon and our observations are striking, but we think that fundamentally different processes are involved for three main reasons: 1- The bends we observed in the filaments were often different from 90°. In the LC, the reorientation was of 180° and even 360° in some cases (see Section S3). 2- In water, the bend at the junction between two different shapes is not only observed when the motor switches to a CW rotation, but also when there is a polymorphic transformation while the motor rotates CW (without any switch)^6^. In the present work, we never observed a reorientation without a CCW–CW switch in agar or in LC solutions. 3- In LC, only half of the CW switches triggered a reorientation of the filament, but the polymorphic transformation should always create a 90° bend. The observation of several cases where the filament keeps its orientation after a CCW–CW switch indicates that the elastic force, created by the LC matrix, is high enough to keep the axis of the filament straight during a polymorphic transformation that would normally create a bend of the filament. Thus, the force created by the bend of the filament seems to be too weak to trigger a reorientation. The force must therefore come directly from the motor and be transmitted to the filament by the locked hook proposed here.

Our characterization of the behavior of bacterial flagella first in soft agar and then in a water-based LC tissue model leads us to conclude that the hook of the bacteria can work in two modes of rotation: the conventional rolling rotation mode and the locked one^3,4^. This process seems necessary to move efficiently in confined environment. Indeed, as previously observed for *V. alginolyticus* ^33^, the capability of the hook to operate in two different modes procures an enhanced motility to bacteria by enabling them to reverse their movement and to escape from obstacles.

An important result is the observation that the hook can become rigid only upon a CCW–CW switch. Interestingly, this asymmetry between the CW–CCW and CCW–CW switches was previously observed in water and when bacteria swarm^6,25,28,34^. When bacteria change their swimming direction in water (tumble), one or few filaments get out of the bundle behind the bacterium, but only after a CCW–CW switch. When the opposite switch (CW–CCW) occurs, the orientation of the filaments does not change (or they change by a much smaller angle), possibly because the hook remains flexible. Furthermore, the reorientation process after a CCW–CW switch in water is the same as described here: the proximal end of the filament changes its direction while the filament does not rotate^6,28^. A recent publication has also shown that CCW–CW switches in a strain with several flagella does not always result in a reorientation of the filament, a result coherent with the fact that CCW–CW switches are necessaries but not sufficient to trigger a locked hook^35^. Finally, swarming bacteria can also make reversal moves, but their analysis is more complex since these bacteria have many filaments and they are in close contact with each other^25^. Nevertheless the steps in the swarming transition are very similar to the motor-driven one described here. In particular, the transition happens only after a CCW–CW switch. These observations all agree with the proposed explanation of a locked hook.

Thus, it seems that the presence of the locked hook is not specific to the LC medium or confined environments, and it might be a general property of the flagellar motor. The locking of the hook is a fundamental capability of flagella that allows bacteria to reorient their movements in confined environments^11^, when they encounter obstacles^9,10^, and we think that it can be generalized to swarming bacteria^25^. It could also play an important role in the standard tumbling process, allowing a larger change of orientation of the filament after a CCW–CW switch.

The conformation change in the hook structure required to make it rigid is not known, but the asymmetry in our observations indicates that the chiral character of the FlgE proteins composing the hook could play an important role. From our measurements, we also conclude that the manifestation of the locked-hook mode seems to be independent of the torque generated by the motor, a useful information for the formulation of a model.

Finally, this study not only shines light on the behavior of bacteria in inhomogeneous and anisotropic environment that closely represent biological tissues, but also demonstrates how LC tissue models can be used as a versatile tool to study the fundamental properties of motile microorganisms. Indeed, the confinement of the movement in one dimension (still in an open 3D environment) greatly simplifies the observations and analysis, allowing a more precise understanding. This illustrates the huge potential of such tissue models, including also the future possibilities of actively controlling the behavior of living microorganisms.

## Methods

### Bacterial culture

The results reported here were obtained using *E. coli* strain HCB1737^29^ (multiple filaments) and *Salmonella* strain EM800^20^ (single filament). A cysteine is incorporated into the filament protein (FliC) of these strains, so that their filaments can be labeled with fluorophores functionalized with maleimide. For the two strains, isolated bacterial colonies were grown on agar plates (15 g agar, 10 g Bacto-Tryptone and 5 g NaCl in 1 liters) at 37 °C for 24 hours. A saturated culture was then made by incubating one colony from these plates in 10 mL of Tryptone broth (10 g Bacto-Tryptone and 5 g NaCl in 1 liters) for 15 hours in a shaking incubator at 200 rpm. To ensure good motility for the *E. coli* strain, 100 *μ*L of saturated culture was diluted in 10 mL of Tryptone Broth and grown to exponential phase at 34 °C for 4 hours in a shaking incubator at 200 rpm. In the case of *Salmonella*, 40 *μ*L of saturated culture was diluted in a solution made with 5 mL of Tryptone Broth and 5 mL of Lysogeny broth (15 g agar, 10 g Bacto-Tryptone, 5 g NaCl and 5 g yeast extract in 1 liters) and grown in the same conditions as *E. coli*.

### Filaments labeling

The final exponential culture was washed once by centrifugation (1500 × *g* for 5 minutes) and then suspended in 100 *μ*L of motility buffer (10 mM KPO_4_, 0.1 mM EDTA and 10 mM lactic acid). 400 *μ*M of AlexaFluor 546 or 488 (Invitrogen A10258 or A10254, Carlsbad, CA) was added to the solution for 3 hours at room temperature to label the filaments. The bacteria were then washed twice by centrifugation and finally suspended in around 3 mL of motility buffer containing 20 mM of glucose to reach an OD_600_ ∼ 1. The glucose was added to ensure continuous bacterial motility when bacteria have consumed all the oxygen in the chamber^19,36^.

### Soft agar solution

To obtain a soft agar solution within our chamber, 180 mg of agar was added to 30 ml of motility buffer and the mix was boiled to obtain a concentration of 0.6%. The temperature of this stock solution was maintained around 90 °C. Before preparing a sample, 1 ml of this solution was cooled for 1 minutes. Then 100 *μ*l of bacteria were added to 100 *μ*l of warm solution (around 45 °C) to obtain a final concentration of 0.3% agar. This solution was then quickly added to the chamber, before it could solidify. To allow the formation of the agar matrix, the sample was cooled down to room temperature (around 20 °C) for 5 minutes before use.

### DSCG LC solution

To make the stock solution of the DSCG LC, 16 wt% of DSCG was added to 84 wt% of motility buffer containing 20 mM of glucose. To ensure complete homogeneity, the solution was heated at 55 °C for 5 minutes and then shaken for 1 minute. The stock solution was kept at 4 °C and used within 2 weeks. Before mixing the stock solution with labeled bacteria, the DSCG solution was heated again at 55 °C for 5 minutes, shaken for 1 minutes and then cooled to room temperature. 25 *μ*L of motility buffer containing 20 mM of glucose and 15 *μ*L of bacterial solution were added to 120 *μ*L of DSCG stock solution to obtain a concentration of 12.2 wt%.

### Chamber

Custom optical chambers were fabricated for our experiments. First, two glass coverslips were coated with PI-150 1% (Nissan Chemical B79032-50, Houston, TX) by spin coating. The PI-150 surface was then rubbed unidirectionally using a technique described in Seo *et al*.^37^. This step was skipped for experiments in the agar solution. To create a small chamber, the two coverslips were fixed together by applying in the periphery UV glue (Norland, Optical Adhesive 65, Cranbury, NJ) containing 60 *μ*m glass microspheres used as spacers (Duke Scientific Corporation 9060, Palo Alto, CA) (see Duchesne *et al*.^38^ for more details). After the insertion of the LC solution in the cavity (by capillarity), the chamber was sealed using transparent nail polish to prevent the evaporation of the fluid.

### Acquisition

Experiments were performed on an IX71 Olympus inverted microscope equipped with an EMCCD iXon3 888 camera (Andor Technology, South Windsor, CT). The X-cite 120LED lamp with a TRITC-A or FITC-3540C filter (Semrock, Rochester, NY) was used to observe fluorescent filaments. To record the rotation of the filaments, a 100x oil objective with 1.4NA (Olympus MPLAPON100XO, Tokyo, Japon) was employed. In agar, to obtain clear images of the filaments while following at the same time the bacteria displacements, 10 s long videos at a frame rate of 193 fps were recorded using a custom Labview program (National Instruments, Austin, TX). The fluorescent light was triggered by the camera to expose the filaments to light only during the exposition time. The videos were manually analyzed to identify the escape and reversal events.

In LC, 10 s long videos at a frame rate between 30 and 40 fps (acquisition time of 33 ms to 25 ms) with an exposure time of 2 ms were recorded. Each filament of the single flagellum strain (EM800) was traced in each frame using a custom Matlab algorithm (MathWorks, Natick, MA), and then manually verified and modified if necessary. The mean translation speed of the filament was calculated for each bacterium using a custom tracking algorithm in Matlab. Finally, the rotation speed of each filament was calculated by applying a Fast Fourier Transform on its positions (see section S2 for more details).

Both in agar and LC samples, the conformation was visually identified before, during and after the occurrence of an event. This identification is necessary to infer the direction of rotation of the filament. Indeed, the identification of the conformation is possible by qualitatively observing the diameter and the pitch of the filament. To verify whether this method was robust, we measured the diameter and pitch of several filaments in agar (movies S1*A*–*E*) and LC solutions (movies S2*A*–*C*, S3*A*–*C* and S4*A*–*C*), and then compared these measurements with the visual identification. As we can see in figure S4, the populations corresponding to each visually identified conformation are well isolated in the diameter vs pitch plot, which indicates a correct identification. Indeed, the different conformations can be easily observed when looking at movies (for examples Figs 2 and 3).

## Electronic supplementary material

Supplementary information

Movie S1A

Movie S1B

Movie S1C

Movie S1D

Movie S1E

Movie S2A

Movie S2B

Movie S2C

Movie S3A

Movie S3B

Movie S3C

Movie S4A

Movie S4B

Movie S4C

Movie S5A

Movie S5C

Movie S6

Movie S7A

Movie S7B

Movie S8
