# Supplementary material for: Transient locking of the hook procures enhanced motility to flagellated bacteria
Source: Sci Rep. 2017 Nov 27;7:16354. doi: 10.1038/s41598-017-16562-4 (PMC5703839; doi:10.1038/s41598-017-16562-4)
Supplement: Supplementary file 1 — Supplementary information [file 41598_2017_16562_MOESM1_ESM.pdf]

Supplementary Information of "Transient locking of the hook procures enhanced motility to flagellated bacteria"

Ismaël Duchesne, Tigran Galstian and Simon Rainville

October 15, 2017

S1 Multi-flagellated *E. coli* strain

By observing a multi-flagellated strain, we confirmed the presence of the locked hook mode and its importance for the reorientation of filaments (see Fig. S1 and movies S5 *A* and *B*). It is worth noting that, unlike what was observed with single flagellated bacteria, the reorientation of a flagellum does not necessarily result in a change of swimming direction (which is determined by the total thrust applied by all the filaments). Indeed, we have often observed a rather spectacular mode of bacterium swimming with one (or few) filaments rotating in front of the body, opposing the cell movement, while most of its flagella are behind its body (see Fig. S2 and movie S6). We emphasize that the "front" filaments never pull the cell body, they only slow it down.

Another particularity of multi-flagellated bacteria is that the filaments in front of the body can flip behind the cell using a completely different mechanism (see Fig. S2 and movie S6). In contrast to the motor-driven

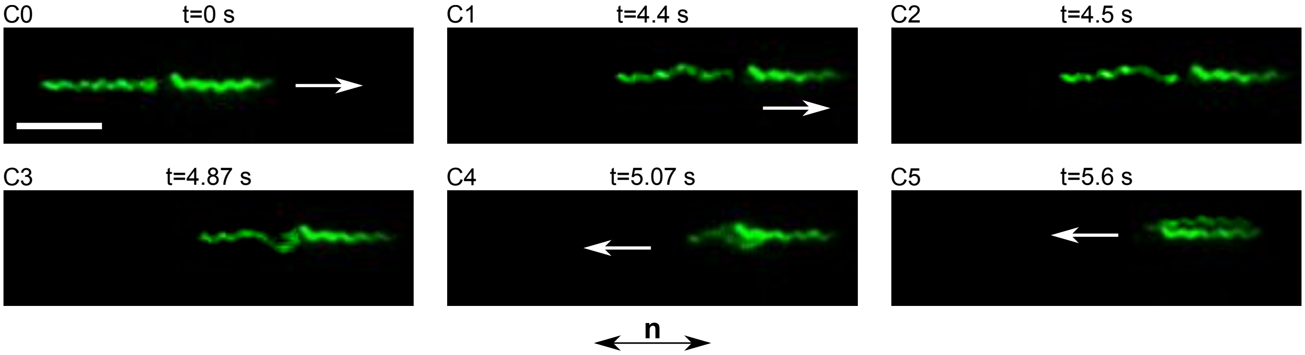

Figure S1: Sequence of images taken from movie S5 that shows a transition for an *E. coli* bacterium. The labels corresponding to the schematics in Fig. 5 of the paper are noted at the top left of each image. The filament in front of the bacterium does not rotate, it is in fact two filaments stuck together. This is an atypical case where the main rotation direction of the motor is CW instead of CCW. Thus, steps C0 and C1 are not the same as those found in Fig. 5 of the paper (see section S3). At step C0, the bacterium runs with its motor rotating CW and a curly filament (curly 1). After a CW−CCW switch, the base of the filament changes its polymorphic conformation from curly to normal. The other steps follow Fig. 5 of the paper. The time stamp of the frame is shown above each image. The white arrows indicate the direction of the displacement of the bacterium's body. When there is no arrow in a box, the cell is at rest. The white bar at the bottom left of the first image measures 5 μm. The bottom arrow with the letter **n** indicates the orientation of the anisotropy axis of the LC.

transitions described in the paper, these "hydrodynamic" flips are driven by the movement of the cell body (pushed by the bundle) and the thrust of the filaments, and there is no switch in the motor's direction of rotation, nor any polymorphic transition involved.

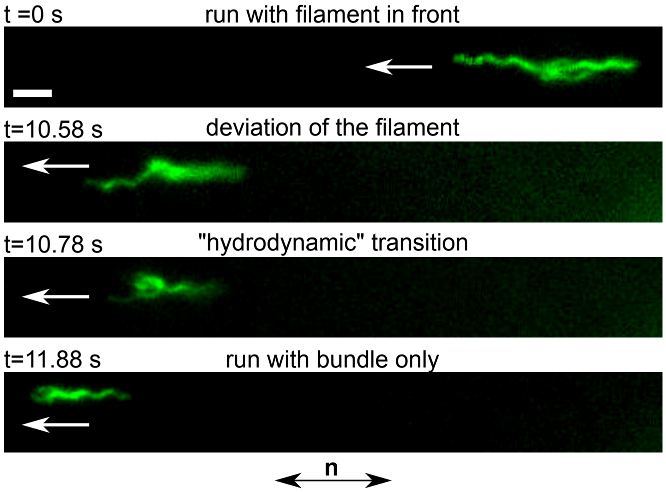

Figure S2: Sequence of images of a bacterium with multiple filaments extracted from movie S6. At $t = 0\,\text{s}$ all the filaments rotate CCW and have a normal form, but one of them is in front of the cell body ($v = 3.0 \pm 0.2\,\mu\text{m/s}$). At $t = 10.58\,\text{s}$, the base of the front filament starts to deviate from the axis of the LC. At $t = 10.78\,\text{s}$ a "hydrodynamic" transition (see text) begins when the body of the bacterium passes in front of the base of the filament. At $t = 11.88\,\text{s}$, the bacterium swims with all its filaments in a bundle behind its body ($v = 8 \pm 1\,\mu\text{m/s}$). The white bar in the top image measures $3\,\mu\text{m}$ and the white arrows show the swimming direction. The bottom arrow with the letter **n** describes the orientation of the anisotropy axis of the LC.

S2 Torque of the filament

Previous work has shown that the torque of the motor can influence its switching dynamics [1]. Since the locking of the hook is tightly associated with switching events, it seems reasonable to think that the torque generated by the motor could have an effect on the triggering of this state. To verify this hypothesis, the torque on the filament was computed and compared between events when the hook locked, and others when it did not. To estimate the torque from measurable quantities, we suppose our medium is a Newtonian fluid and estimate the shape of the bacterium by a prolate spheroid with semiminor axis $a = 0.5\,\mu$m and semimajor axis $b = 1.5\,\mu$m. Using the Purcell's model, we obtain the following expressions for the torque

$$N_f = -Bv + D\omega. \tag{S1}$$

The parameters B and D can be calculated from

$$B = k_n L \frac{\lambda}{2\pi} \sin\Psi \tan\Psi (1-\gamma)\eta, \tag{S2}$$

$$D = k_n L \left(\frac{\lambda}{2\pi}\right)^2 \sin\Psi \tan\Psi (1+\gamma\cot^2\Psi)\eta, \tag{S3}$$

with

$$k_n = \frac{8\pi}{2\ln\frac{c\lambda}{r}+1} \tag{S4}$$

and

$$k_t = \frac{4\pi}{2\ln\frac{c\lambda}{r}-1}. \tag{S5}$$

Here, v is the speed of the bacterium, ω is the angular speed of the filament, $\Psi = 41\,^\circ$ is the helix angle made by the filament with its axis, $\gamma = \frac{k_t}{k_n}$, $r = 20\,$nm is the estimated radius of the tube that composes the filament, and $c = 2.4$ is the Lighthill constant [2–4]. In addition, the length of the filament (L) was measured from our videos, the viscosity is $\eta = 60 \pm 1\,\mathrm{mPa\cdot s}$ [4], the pitch of the filament (λ) is $2.2\,\mu$m for the normal conformation, and $1.1\,\mu$m for the curly 1 filaments [5,6].

As we can see, we need to measure the speed of the bacterium and the angular speed of the filament to calculate the torque. To measure the angular speed, we traced the position of the filament in every frame of our movies where the filament was sharp (see Fig. S3**a** and movie S8). This step was first performed automatically, and then verified to manually correct the traces if needed. The translation speed of the bacterium was calculated from the mean position of the trace in each frame. The filament's traces from each frame were then all centered at the same point and rotated to orient them parallel to the X axis. For each vertical line in the images, we obtained the vertical position of the trace vs time. As we can see in Fig. S3**b**, a superposition of two oscillations at two different frequencies is clearly visible. A Fast Fourier Transform (FFT) of the vertical position vs time revealed two main peaks in the spectrum, one corresponding to the (slow) rotation of the body, and the other one to the (fast) rotation of the filament (see Fig. S3c). To precisely identify the two angular speeds, a Gaussian fit was performed around the two highest peaks in the spectrum.

Using the measured speeds and the Purcell model described above, we calculated the torque of the filament one second before each CCW−CW switch. We separated cases A and B from case C (see Fig. 5 of the paper) to compare the events involving a locking of the hook, and those that do not. The torque just before the events with and without a locked hook was respectively $4500 \pm 300\,\mathrm{pN\cdot nm}$ and $5300 \pm 600\,\mathrm{pN\cdot nm}$. These values are consistent with our previous work [4]. We conclude that the torque is not significantly different in these two cases, and therefore that the locking probability of the hook is independent of the torque.

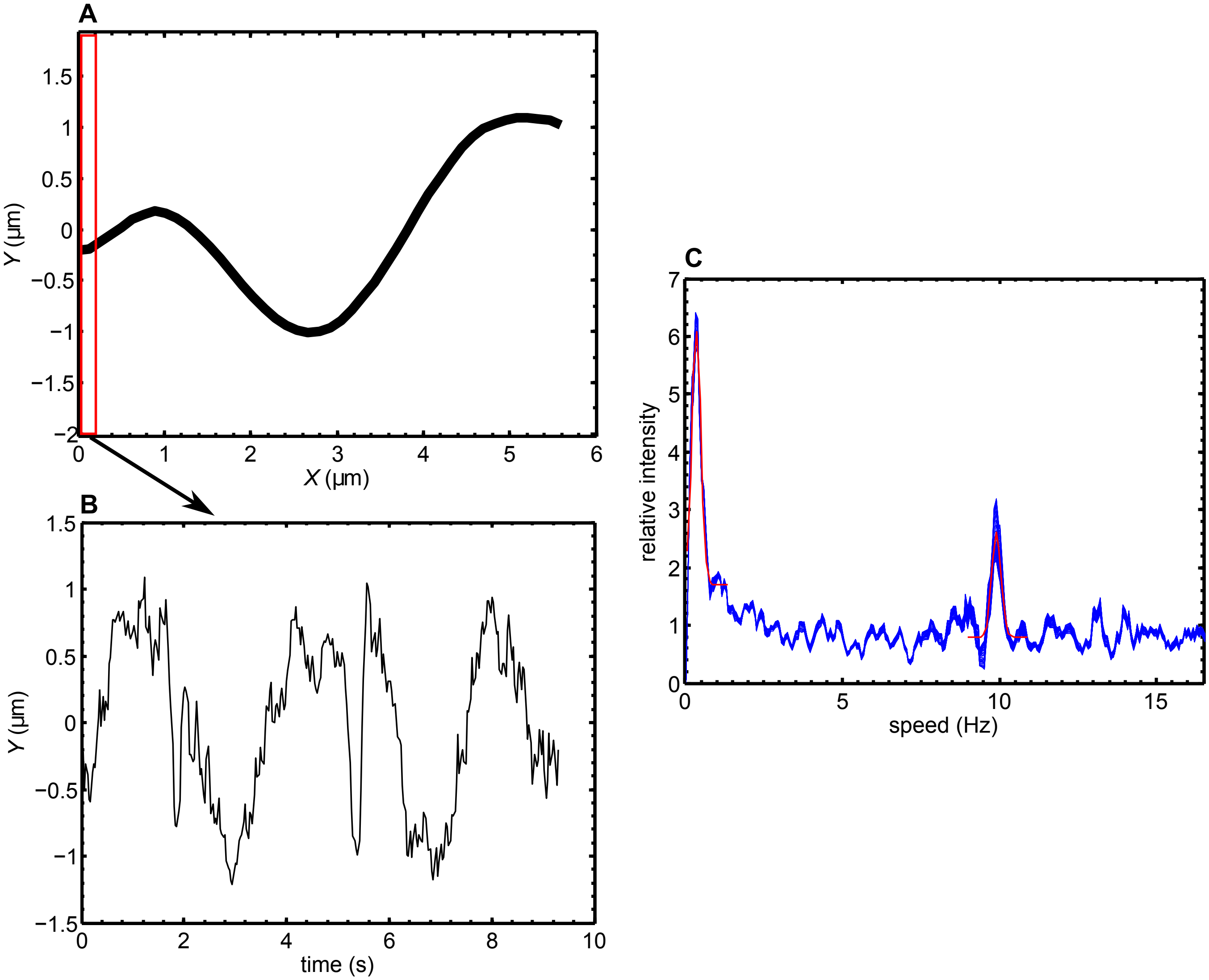

Figure S3: Characterization of the rotation of one filament. (**A**) First frame of the trace of the filament in movie S8. (**B**) Position in the Y axis of the segment contained in the red box in the figure **A** versus time. (**C**) FFT of the position in the Y axis versus time for each X position. The two red lines in C represent the best Gaussian fit around the two frequencies where the FFT has maximums.

S3 Atypical cases

In Fig. 5 of the paper, we have shown three typical cases observed after a CCW−CW switch. However in approximately 5% of cases, the behavior of the bacteria was different from these typical cases. We have seen 7 occurrences of transitions where the filament complete a full turn (see movies S7 *A* and *B*). This transition was similar to the case B (incomplete transition) except that the reorientation of the filament was about $360°$ instead of $\leq 90°$ (in step B3). These cases were counted as case B. The second and third atypical cases are very similar to the case A so they were counted as that case in the paper. The second case happens when the main rotation state of the motor is CW instead of CCW. This case is similar to A, except that the bacteria skip steps A1 and A6. Before a transition occurs, there is a short period of time where the motor rotates CCW during which the filament is partially or completely in the normal conformation (see Fig. S1). The third case is exactly the same as A, but during the winding of the filament (step A4), there is a CW−CCW switch. When this event occurs, the reorientation continues as for the conventional case but the filament changes its polymorphic conformation from curly to normal.

S4 Additional figure

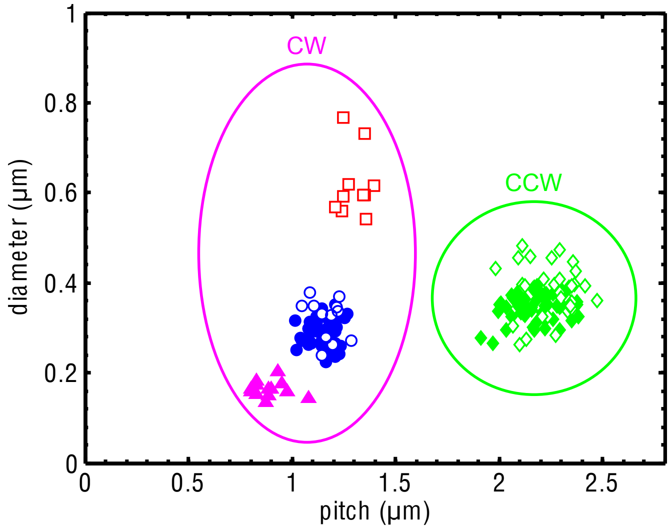

Figure S4: Determination of the rotation direction of the filament by using measures of diameter and pitch. Measures taken from agar movies (S1 *A*-*E*) are reported with empty symbols and those taken from LC movies (S2 *A*-*C*, S3 *A*-*C* and S4 *A*-*C*) with filled symbols. Each symbol corresponds to a specific conformation that we have visually identified : ○ = curly 1, △ = curly 2, □ = semi-coiled and ◊ = normal. Data in the region enclosed by the magenta line represent filaments that rotate CW, and those in the region enclosed with the green line, represent those that rotate CCW.

References

[1] Bai, F., Minamino, T., Wu, Z., Namba, K. & Xing, J. Coupling between switching regulation and torque generation in bacterial flagellar motor. *Phys. Rev. Lett.* **108**, 178105 (2012). URL. http://link.aps.org/doi/10.1103/PhysRevLett.108.178105.

[2] Chattopadhyay, S., Moldovan, R., Yeung, C. & Wu, X. L. Swimming efficiency of bacterium *Escherichia coli*. *Proc. Natl. Acad. Sci. USA* **103**, 13712–13717 (2006). URL. http://www.pnas.org/content/103/37/13712.abstract.

[3] Martinez, V. A. et al. Flagellated bacterial motility in polymer solutions. *Proc. Natl. Acad. Sci. USA* **111**, 17771–17776 (2014). URL. http://www.pnas.org/content/111/50/17771.abstract.

[4] Duchesne, I., Rainville, S. & Galstian, T. Bacterial motility reveals unknown molecular organization. *Biophys. J.* **109**, 2137–2147 (2015). URL. http://www.sciencedirect.com/science/article/pii/S0006349515010073.

[5] Turner, L., Ryu, W. S. & Berg, H. C. Real-time imaging of fluorescent flagellar filaments. *J. Bacteriol.* **182**, 2793–2801 (2000). URL. http://jb.asm.org/content/182/10/2793.short.

[6] Hasegawa, K., Yamashita, I. & Namba, K. Quasi-and nonequivalence in the structure of bacterial flagellar filament. *Biophys. J.* **74**, 569–575 (1998). URL. http://www.sciencedirect.com/science/article/pii/S0006349598778154.
